# A Water-Free Omega-3 Fatty Acid Eye Drop Formulation for the Treatment of Evaporative Dry Eye Disease: A Prospective, Multicenter Noninterventional Study

**DOI:** 10.1089/jop.2021.0102

**Published:** 2022-06-09

**Authors:** Christina Jacobi, Simone Angstmann-Mehr, Anja Lange, Thomas Kaercher

**Affiliations:** ^1^ AUGEN & HAUT Zentrum—Praxis Dr. Jacobi, Nürnberg, Germany.; ^2^ Novaliq GmbH, Heidelberg, Germany.; ^3^ Augenarztpraxis, Heidelberg, Germany.

**Keywords:** dry eye disease, omega-3 fatty acid, PMCF, perfluorohexyloctane, keratoconjunctivitis sicca, DED

## Abstract

**Purpose:**

NovaTears^®^+Omega-3 is a water-free eye drop solution with non-animal-derived omega-3 fatty acids. It allows to supplement omega-3 fatty acids directly in the tear film of patients with dry eye disease (DED). This post-market clinical follow-up (PMCF) study evaluated for the first time the effects on clinical signs and patient symptoms of DED, and safety and tolerability of NovaTears+Omega-3 (0.2%) eye drops, when used in accordance with its approved label.

**Methods:**

A prospective, multicenter, single-arm, uncontrolled, open-label observational cohort study was performed in patients suffering from symptoms of evaporative DED. Patients were treated 4 times daily bilaterally according to the instructions for use for 8 weeks, and standard of care clinical end points were assessed at baseline and follow-up. The trial was conducted at 2 investigational sites in Germany, Europe.

**Results:**

Thirty-six patients were included and 33 completed the study. NovaTears+Omega-3 (0.2%) showed clinically and statistically significant improvements in various clinical signs, such as total corneal staining, tear film break-up time, and Meibomian gland dysfunction (MGD) score, as well as in symptoms measured by Ocular Surface Disease Index (OSDI^©^) and visual analog scales over the 8-week treatment period with change from baseline *P* values all <0.0001. No worsening of any safety parameter (intraocular pressure, slit-lamp examination, visual acuity) was observed, and no adverse event was reported throughout the study.

**Conclusions:**

In this observational PMCF study, NovaTears+Omega-3 was safe and well tolerated. Treatment over an 8-week period resulted in significantly improved clinical signs and subjective symptoms in patients with evaporative dry eye. The study was registered at www.clinicaltrials.gov (NCT04521465).

## Introduction

Dry eye disease (DED) is one of the most common ocular surface disorders with more than 85% of affected patients suffering from evaporative DED.^1^ Evaporative DED is caused by excessive tear evaporation due to an abnormal tear film lipid layer, or dysfunction of the Meibomian glands. Meibomian glands line the upper and lower margins of the eyelids and are the sole natural source for tear lipid production. Excessive tear evaporation is linked to an inadequate tear lipid layer and may act as trigger for DED.^2^ For evaporative DED treatment, options are currently limited. Physical therapies, such as eyelid hygiene, warm compresses, intense pulsed light (IPL), or thermal pulsation system (eg, LipiFlow™), aim to increase lipid outflow, whereas lipid containing artificial tears aim to substitute the lipid layer.^3^

In recent years, the role of essential fatty acids (EFAs) in DED has been explored. Omega-3 fatty acids are essential polyunsaturated fatty acids, to be obtained from external sources because humans are unable to synthesize them *in vivo*.^4^ There is evidence that omega-3 fatty acids are an important natural component of the human tear film. A reduction of omega-3 content in the tear film is correlating with DED.^5^ Studies indicate that dry eye patients with Meibomian gland dysfunction (MGD) show a decrease of polyunsaturated fatty acids in the tear film.^6–8^ Omega-3 fatty acids have antioxidant and anti-inflammatory properties^9^ and may help mitigating DED manifestation.

Hence, several clinical studies addressing the efficacy of daily oral supplementation with EFAs on clinical measures of DED have been performed. As of to date, these studies revealed mixed results^10^ providing inconsistent evidence for the therapeutic benefit of oral supplementation with omega-3. In the recent DREAM study, a clinical benefit of oral supplementation with omega-3 fatty acids was not demonstrated in ∼350 patients.^11^ Thus, despite clear correlation of tear film deficiency of omega-3 and DED manifestation, oral supplementation of omega-3 is under discussion for the treatment of this disorder.

It remains further unclear if and in what amount orally supplemented omega-3 can reach the ocular surface. Therefore, the concept of topical instillation of omega-3 fatty acids through a classical eye drop is a promising new approach to overcome the obvious limitations of oral supplementation.

Due to their lipophilic nature, omega-3 fatty acids do not mix well in aqueous eye drop formulations, thereby limiting their development. Water-based eye drops generally require the addition of preservatives to prevent oxidation of the omega-3 fatty acids and the addition of emulsifiers to dissolve them. Novaliq's EyeSol^®^ technology is a water-free technology for ophthalmic products. It significantly increases the residual time of the eye drops on the ocular surface and overcomes the above-described limitations of water-based eye drops: lipophilic substances are well dissolved and the resulting clear solution does not require addition of preservatives. Of additional advantage is the low viscosity, a very similar refractive index to water and the smaller drop size, which does not stimulate the natural reflex blinking.

NovaTears^®^+Omega-3 is a class IIb medical device, which is Conformitè Europëenne (CE) marked since 2017 and commercially available in Europe (brand name EvoTears^®^ Omega), Australia, and New Zealand. It contains perfluorohexyloctane, a semifluorinated alkane, and 0.2% docosahexaenoic acid, an omega-3 fatty acid derived from algae.

NovaTears+Omega-3 addresses evaporative dry eye via its novel physicochemical mode of action. The low surface tension of the main component perfluorohexyloctane ensures (1) rapid spreading across the entire ocular surface, (2) long-lasting interaction with the lipids of the outer tear film layer, and (3) topical substitution of omega-3 directly into the tear film. Thus, NovaTears+Omega-3 stabilizes the tear film layer, prevents excessive evaporation of the aqueous tear film components, and likely protects the ocular surface from noxious oxidative processes.

The aim of the present post-market clinical follow-up (PMCF) study was to confirm the efficacy of NovaTears+Omega-3 (0.2%) eye drops in relieving patients' subjective symptoms and improving the clinical signs of DED, and to collect data supporting safety and tolerability.

## Methods

### Study design

This was a prospective, multicenter, uncontrolled, open-label observational PMCF study designed to evaluate the efficacy, safety, and tolerability of NovaTears+Omega-3 (0.2%) eye drops, when used topical in accordance with its approved labeling.

The study was performed at 2 investigational sites in Germany (Heidelberg and Nürnberg) and was reviewed and approved by the Ethics Committee of Baden-Württemberg in Stuttgart, Germany. The study was registered at www.clinicaltrials.gov and was conducted in accordance with the observational plan and the ethical principles that have their origins in the Declaration of Helsinki and all other applicable regulatory requirements and laws.

All patients had to provide written informed consent before study enrollment or the conduct of any study-related procedures. After informed consent was obtained, patients who met all eligibility requirements were treated with NovaTears+Omega-3 bilaterally 4 times daily according to the instructions for use (IFU) for a duration of 8 weeks. Standard of care clinical assessments were conducted at baseline and at 8 weeks (±5 days) of follow-up. The baseline assessments were carried out in the framework of a regular visit to diagnose the disease and/or to evaluate severity of disease and/or to evaluate changes to previous visits. During the second visit, the clinical progress under a new treatment was monitored, which is also a routine procedure after ∼2 months of therapy.

### Patients

Thirty-six patients older than 18 years with a patient-reported history of DED in both eyes were enrolled competitively at both investigational sites. Patients were selected based on the relevant product labeling in the IFU. All patients deemed appropriate for treatment with NovaTears+Omega-3 according to the IFU were asked by the investigators to participate in this PMCF study.

Besides from the selection according to the IFU, patients had to fulfill the following main inclusion criteria: history of DED for at least 6 months, total corneal fluorescein staining (tCFS) ≤11 [National Eye Institute (NEI) scale], MGD score ≥3, tear film break-up time (TBUT) ≤8 s, Ocular Surface Disease Index (OSDI^©^) score ≥25, and Schirmer's test I score ≥5 mm. Patients should have applied eyelid hygiene for at least 14 days before enrollment and be willing to continue during the study.

Patients were excluded if they had a known allergy or sensitivity to the medical device or its components, ocular surface pathology, and/or clinically significant slit-lamp findings requiring prescriptive medical treatment. Patients may not have used any pharmacological topical ocular medication within 30 days or lipid containing eye drops within 15 days prior visit 1. Procedures affecting Meibomian glands were not allowed within 6 months prior enrollment.

Patients with DED that in the opinion of the investigator was considered not to respond to topical treatments, for example, secondary to scarring, irradiation, alkali burns, cicatricial pemphigoid, destruction of conjunctival goblet cells, or abnormal lid anatomy were excluded. Oral medications known to cause ocular drying were not allowed if on a nonstable regimen within 30 days prior visit 1 or during the trial. Patients using contact lenses were also not allowed to participate.

Eyes were eligible for analysis if they met all the inclusion criteria and none of the exclusion criteria. Both eyes were treated, and for cases in which both eyes were eligible, the right eye was evaluated as “study eye.”

### Assessments of outcome measures

During the study, the following efficacy assessments of signs were performed at baseline and 8 weeks follow-up: tCFS, assessed in both eyes using the NEI scale which ranges from 0 to 3 for each of the 5 areas of the cornea (higher values describe greater staining and corneal damage), TBUT analysis, Schirmer's test I without anesthesia, and Meibomian gland assessment [MGD score evaluation by Korb Meibomian Gland Evaluator™ (MGE)]. For the latter, 5 central glands on lower eyelid were evaluated, each was scored from 0 to 3; 0 = normal; 1 = thick/yellow, whitish, particulate; 2 = paste; 3 = none/occluded; the total MGD score ranged from 0 to 15.

Symptom assessments were performed using visual analog scales (VAS) ranging from 0 to 100, with 0 = no discomfort and 100 = maximal discomfort for the following symptoms: severity of dryness, burning/stinging, sticky feeling, foreign body sensation, itching, blurred vision, sensitivity to light, and pain. In addition, frequency of dryness and awareness of dry eye symptoms were assessed using VAS ranging from 0 to 100 scale, with 0 = never and 100 = all the time. Symptoms were also assessed via the OSDI questionnaire, a composite end point built on 12 questions with a total score ranging from 0 to 100, with higher scores representing a worse condition.

Furthermore, for the evaluation of the readability of the IFU, patients were asked to answer 4 questions on a readability questionnaire at baseline visit, and for the evaluation of drop comfort and patient satisfaction, patients were asked to answer 5 questions via a score on a VAS after drop instillation at the follow-up visit.

Safety assessments included best corrected visual acuity using Snellen eye chart, slit-lamp biomicroscopy, intraocular pressure (IOP), and ocular and non-ocular adverse events (AEs). Patients were asked during the informed consent process and at the end of each visit to report any AE to the investigator.

### Statistical methods

No formal sample size calculation or formal hypothesis testing has been performed. NovaTears+Omega-3 (0.2%) is a new medical device, and there were no systematic clinical data in the approved indication available to allow an estimate of the effect size on adequate clinical end points under investigation.

A cohort of 30 patients was considered sufficiently large to obtain estimates of performance and safety parameters. From a statistical perspective, this sample size allows to detect a difference between baseline and 8 weeks on a magnitude of 1 standard deviation of intraindividual differences with a power close to 100%, differences of 2/3 of a standard deviation with a power >90%, and differences on a magnitude of half a standard deviation with a power close to 80% (75.4%), assuming a normal distribution of intraindividual differences and a 2-sided type I error of 5%. A previous study with the Medical Device NovaTears with 30 patients demonstrated benefits of the product in several clinical end points.^12,13^

All baseline and end point variables were analyzed descriptively according to a statistical analysis plan. The safety set includes all patients who took any study treatment. The full analysis set (FAS) consists of all patients who completed the 8-week observation period, regardless of whether the patient was compliant with the requested time window. The per-protocol set comprises all patients in the FAS who have completed the study without major protocol deviations and is equal to the FAS in this study.

Protocol deviations were classified before database lock. A total of 4 protocol deviations were recorded for the study and were related to pretreatment, out of window study visits and omission of a questionnaire. All deviations were classified as minor as they do not impact safety, efficacy, or data integrity.

## Results

### Subject disposition and demographics

Thirty-six patients have been screened, and all were enrolled into the study. The first patient started in November 2020 and the last patient left the study in February 2021. From the 36 patients enrolled, 3 patients terminated early. One patient withdrew consent the day after enrollment and did not take any study medication. This patient was excluded from the analysis. Two patients withdrew consent and did not return to visit 2, thus were excluded from the FAS.

The mean age in the FAS population was 54.8 ± 17.86 years. Of the 33 patients, 23 (69.7%) were female and 10 (30.3%) were male. Patients' demographic characteristics are displayed in Table 1.

**Table 1.** Demographics (Full Analysis Set)

	Center 1 (*n* = 18)	Center 2 (*n* = 15)	All subjects (*N* = 33)
Age (years)			
Mean (SD)	63.5 (14.38)	44.5 (16.32)	54.8 (17.86)
Range	23–78	25–76	23–78
Sex, *n* (%)			
Female	15 (83.3)	8 (53.3)	23 (69.7)
Male	3 (16.7)	7 (46.7)	10 (30.3)
Sign and symptom baseline characteristics			
Schirmer's test I		15.6 (7.9)	
MGD score		7.1 (3.3)	
tCFS		5.0 (2.0)	
TBUT		5.76 (1.85)	
OSDI score		51.1 (16.2)	
Dryness score		60.5 (19.5)	
Awareness of dry eye symptoms		52.0 (21.1)	

TBUT, tear film break-up time; MGD, Meibomian gland dysfunction; OSDI, Ocular Surface Disease Index; SD, standard deviation; tCFS, total corneal fluorescein staining.

### Efficacy

**FIG. 1.**
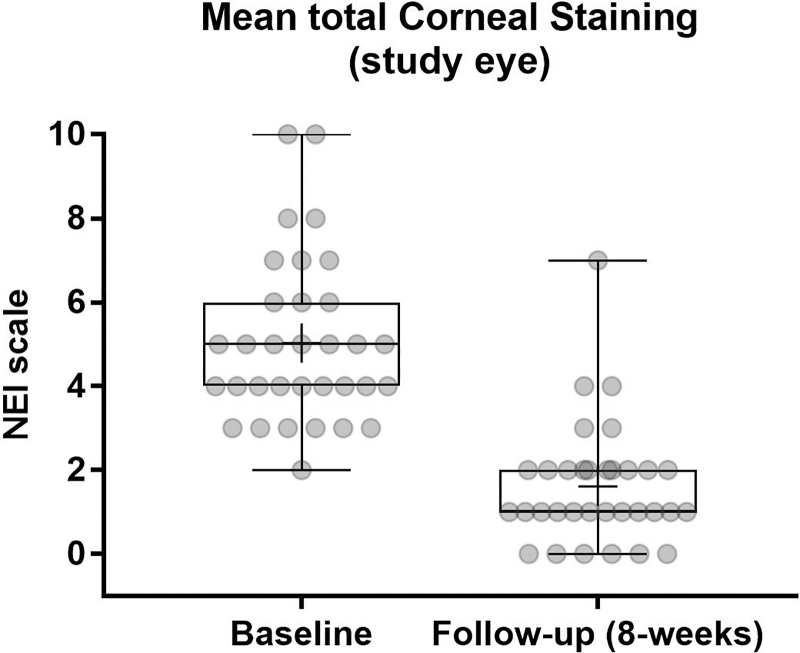
Mean tCFS at baseline and week 8. High statistically significant difference for CFB in tCFS at week 8 (*P* < 0.001). The NEI scale divides the cornea into 5 regions. The total score is the sum of all regions (0–3 per region, total score of 15 indicates maximum staining). CFB, change from baseline; NEI, National Eye Institute; tCFS, total corneal fluorescein staining.

**FIG. 2.**
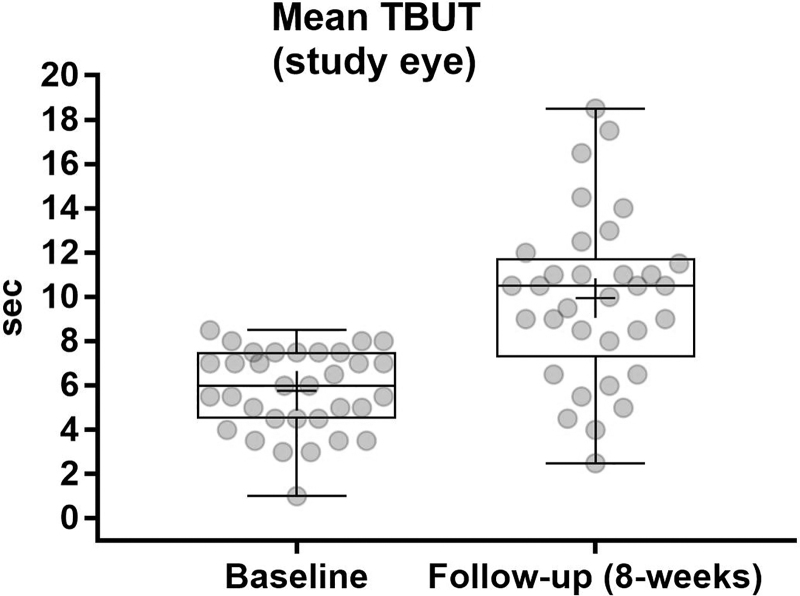
TBUT over the treatment period. Difference for CFB at week 8 (*P* < 0.0001). TBUT, tear film break-up time.

**FIG. 3.**
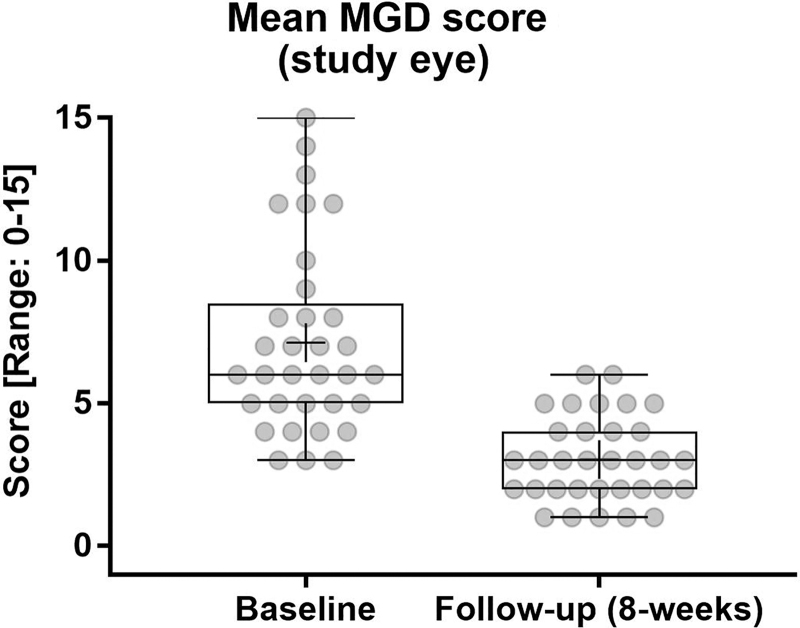
MGD score over treatment period. High statistically significant difference for CFB at week 8 (*P* < 0.0001). Five central glands on lower eyelid were evaluated, each scored from 0 to 3; 0 = normal; 1 = thick/yellow, whitish, particulate; 2 = paste; 3 = none/occluded; the total MGD score ranged from 0 to 15. MGD, Meibomian gland dysfunction.

The tCFS score decreased statistically significant from 5.0 ± 2.0 to 1.6 ± 1.4 after the treatment period with a change from baseline (CFB) of −3.4 ± 2.1 (*P* < 0.0001) (Fig. 1). TBUT increased statistically significant from 5.76 ± 1.85 to 9.94 ± 3.76 with a CFB of 4.18 ± 2.77 (*P* < 0.0001) (Fig. 2). Statistically significant improvements were also observed for the MGD score with a baseline mean of 7.1 ± 3.3, which decreased to 3.0 ± 1.5 at week 8 with a CFB of −4.1 ± 3.3 (*P* < 0.0001) (Fig. 3).

**FIG. 4.**
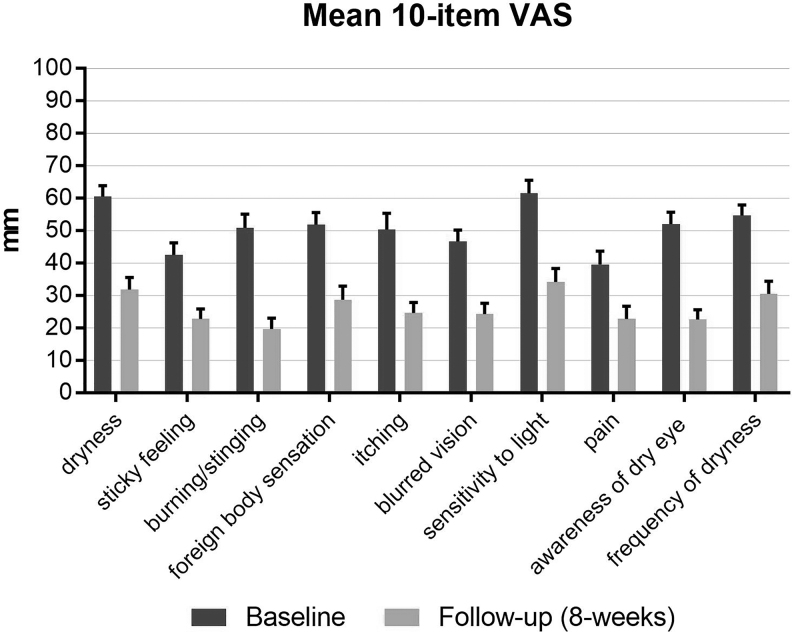
Mean change from baseline (±SEM) for VAS dryness symptoms over the treatment period. High statistically significant difference for CFB at week 8 (*P* < 0.0001). SEM, standard error of the mean; VAS, visual analog scale.

**FIG. 5.**
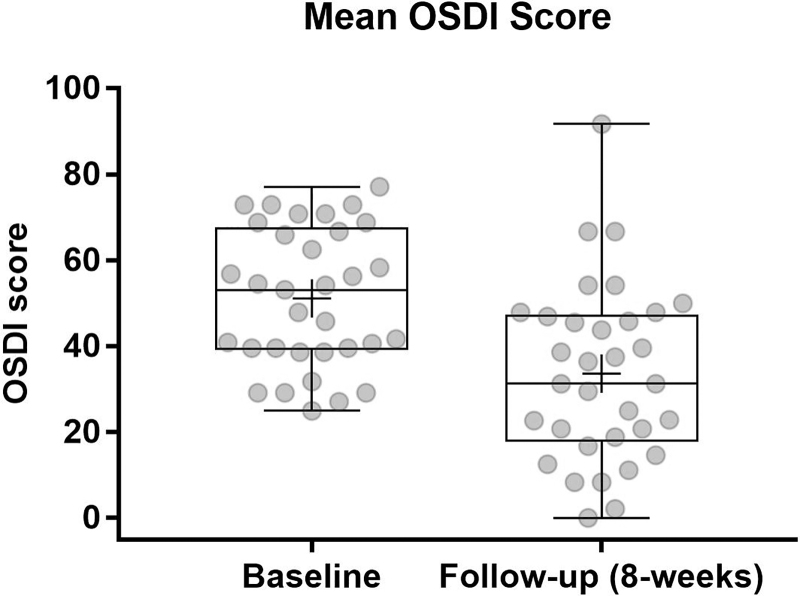
OSDI score over treatment period. High statistically significant difference for CFB at week 8 (*P* < 0.0001). OSDI, Ocular Surface Disease Index.

The study also showed a significant improvement in a broad spectrum of dryness symptoms, as measured by 10-item VAS (Fig. 4) and OSDI scores (Fig. 5). Total OSDI score improved statistically significant from 51.1 ± 16.2 at baseline to 33.6 ± 20.6 at follow-up, with a clinically remarkable reduction by 17.5 ± 20 points (*P* < 0.0001).

For several symptoms on the 10-item VAS, average improvements of more than 50% from baseline were observed: burning/stinging value of 50.9 ± 24.3 decreased by 31.2 ± 28.0 to 19.7 ± 19.2 at follow-up and itching decreased from 50.4 ± 28.7 at baseline by 25.8 ± 28.9 to 24.6 ± 19.0 at follow-up. Awareness of dry eye symptoms decreased from 52.0 ± 21.1 at baseline by 29.4 ± 27.5 to 22.6 ± 17.3 after the treatment period.

**FIG. 6.**
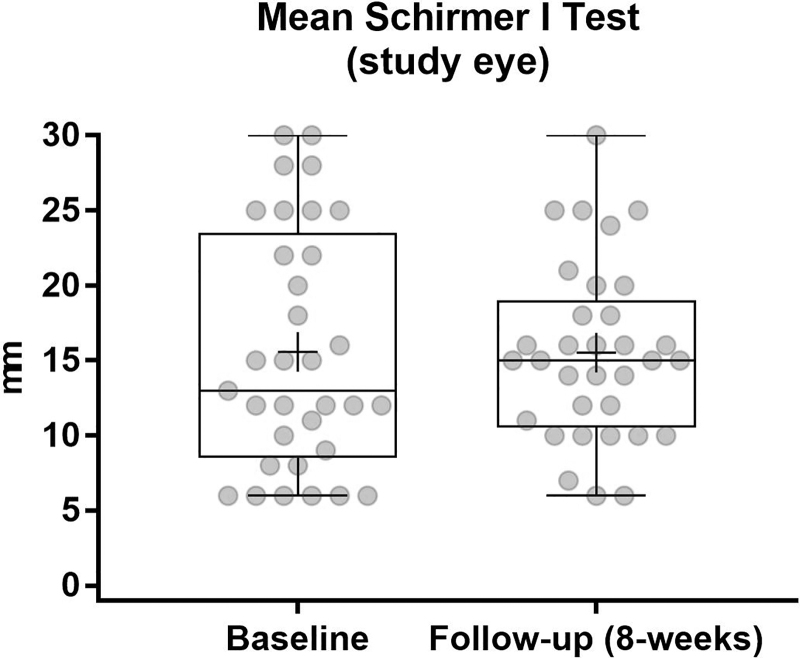
Schirmer's test I over treatment period. Scores remain nearly the same as expected for a population with evaporative DED and normal scores at baseline. DED, dry eye disease.

The Schirmer's scores remained essentially unchanged during the treatment period with a baseline value of 15.6 ± 7.9 and 15.5 ± 5.8 at follow-up (Fig. 6).

In addition, it is of note that slit-lamp examinations after the treatment period revealed improvements of redness/injection of the conjunctiva in 14 patients and the lid in 2 patients, keratitis superficialis punctata in 6 patients, and MGD in 2 patients at the follow-up visit.

Although no formal statistical analyses were performed on efficacy parameters related to potential gender or site effects, there were no apparent differences detected.

### Further assessments

For the evaluation of the drop comfort and patient satisfaction, patients were asked at the follow-up visit to instill the drops onsite and answer 5 questions. For each question, a score on a VAS ranging from 0 to 10 should be selected. No statistical analysis was applied to this parameter. Overall, the ratings were positive, in particular overall satisfaction with the treatment was rated in average with 7.9 ± 2.5. Twenty-six of 32 patients scored between 7 and 10, and only 6 patients scored between 2 and 5.

For the evaluation of the readability of the instruction for use, the patients were asked to read the IFU at visit 1 and to answer 4 questions. For the first 3 questions, patients were allowed to score between 0 and 10 on a VAS. Patients regarded the font size sufficient and the structure clear and understandable with mean scores between 6.8 ± 2.4 and 7.4 ± 1.6. Question 4 was to check if the patient read and understood the IFU correctly. Thirty-one of 35 patients ticked the right answer.

### Safety

There were no AEs reported during the study.

CFB in the mean visual acuity from 0.873 ± 0.217 to 0.868 ± 0.234 at follow-up are regarded minor and are reflecting normal variations. IOP remained essentially unchanged with a CFB of −0.6 ± 1.7 from a mean baseline value of 14.2 ± 1.4 and 13.5 ± 1.7 at follow-up. The slit-lamp examination did not indicate any worsening of a pre-existing finding or a new finding during the cause of the study. In contrast, some of dry eye-related findings disappeared or improved during the cause of the study.

## Discussion

NovaTears+Omega 3 is a water-free eye drop formulation combining perfluorohexyloctane, which is known to reduce signs and symptoms of dry eye,^12,13^ with omega-3 fatty acid esters, which are a natural component of the tear film and potentially important for evaporation and oxidative stress resistance.^9,14^

This multicenter, prospective, open-label observational PMCF study was designed to assess efficacy and safety of NovaTears+Omega-3 in patients suffering from symptoms of evaporative DED. Patients were treated for a period of 8 weeks according to the IFU, and standard of care clinical end points were assessed at baseline and at follow-up.

The patient population included in the study was on average about 55 years old with 70% being female, which is a typical demographic distribution for a DED population.^15^ The mean baseline characteristics of the population (OSDI of about 50; MGD scores of about 7; normal Schirmer values >15 mm/5 min, and tCFS scores of ∼5 based on the NEI score) correspond to a mild-to-moderate evaporative DED population.

The results of this PMCF study show that NovaTears+Omega-3 was safe and efficacious in relieving symptoms and improving signs of DED. The great majority of tested clinical signs and patient subjective symptoms improved statistically significant from baseline. The 2 clinical signs, tCFS and MGD, improved on average more than 50% from baseline, which is considered a clinically meaningful improvement.

For several patient-reported symptoms on the 10-item VAS, average improvements of more than 50% from baseline were observed.

In the present clinical study, patients with an OSDI score >25 were included and a mean improvement by 17.5 points from baseline was seen after 8 weeks of treatment. This is significantly larger than the improvement of 7.3–13.4 points that was regarded as clinically meaningful by Miller et al. in a population of symptomatic patients with an OSDI score >33.^16^

The high patient satisfactory score further underlines the results.

As expected, Schirmer's test did not show improvements. In this study, the average of Schirmer's test at baseline was about 15 mm/5 min, which is in the normal range and not pathologic. This test is a common clinical test for limited tear production, not tear evaporation. In patients with evaporative DED, limited tear production is not the primary issue; it is rather the quality of the tear film and the stability of the lipid layer that leads to fast evaporation of the tear fluid.

No AEs were reported during the trial, and no other safety parameter indicates any deterioration.

This clinical study shows that NovaTears+Omega 3 is safe, well tolerable, and effective in treating both clinical signs and subjective symptoms of DED.

For evaporative DED, lid hygiene, such as warm compresses and lid massage, is an effective base therapy but often associated with limited compliance.^3^ In-office procedures such as LipiFlow and IPL apply the same therapeutic concept using eyelid warming and massage. The second treatment pillar are lipid containing artificial tears or night gels to replenish the lipid layer of the tear film either alone or in conjunction with physical therapies.^3^ Today, dietary supplementation of omega fatty acids is still widely used despite the inconsistency of clinical evidence for systemic application routes.^5,10,11,17^ Escalation therapies include topical azithromycin, topical steroids (short term), as well as topical cyclosporine.^3,17^

The topical application of NovaTears+Omega 3, which combines the anti-inflammatory omega-3 fatty acids with the anti-evaporative properties of perfluorohexyloctane, that has already clinically proven its safety and efficacy,^12,13^ might be an innovative alternative for artificial tears and dietary supplementation for patients with evaporative DED.

There are a few limitations of this observational study: the study was exploratory, without control group, and comparisons were primarily made in comparison to baseline. This implies that potential study effects cannot be fully differentiated from the treatment effect. The other limitation is the rather small sample size of 33 patients. Nevertheless, the study achieved highly significant results relevant for patients and their health care providers.

## Conclusions

This study has demonstrated that NovaTears+Omega-3 with its unique mode of action improves a number of clinically relevant signs and symptoms statistically significantly. The magnitude of improvements was considered clinically relevant, and the product was found to be safe and well tolerated. Supplementing the tear film topically with omega-3 fatty acids presents a new treatment modality and complements the existing treatment armamentarium of DED.
